# Interactions of 17β-Hydroxysteroid Dehydrogenase Type 10 and Cyclophilin D in Alzheimer's Disease

**DOI:** 10.1007/s11064-020-02970-y

**Published:** 2020-01-29

**Authors:** Zdenka Kristofikova, Tomas Springer, Erika Gedeonova, Adéla Hofmannova, Jan Ricny, Lenka Hromadkova, Martin Vyhnalek, Jan Laczo, Tomas Nikolai, Jakub Hort, Tomas Petrasek, Ales Stuchlik, Karel Vales, Jan Klaschka, Jiri Homola

**Affiliations:** 1 National Institute of Mental Health, Topolova 748, 250 67 Klecany, Czech Republic; 2 Institute of Photonics and Electronics of the Czech Academy of Sciences, Chaberska 57, 182 51 Prague, Czech Republic; 3 Department of Neurology, Memory Disorders Clinic, 2nd Faculty of Medicine, Charles University in Prague and Motol University Hospital, V uvalu 84, 150 06 Prague 5, Czech Republic; 4 Institute of Physiology of the Czech Academy of Sciences, Videnska 1083, 142 20 Prague, Czech Republic; 5 Institute of Computer Science, Czech Academy of Sciences, Pod vodarenskou vezi 271/2, 182 07 Prague, Czech Republic

**Keywords:** Mitochondrial matrix proteins, Amyloid β, Transgenic rat model, Alzheimer's disease, Frontotemporal lobar degeneration, Cerebrospinal fluid

## Abstract

The nucleus-encoded 17β-hydroxysteroid dehydrogenase type 10 (17β-HSD10) regulates cyclophilin D (cypD) in the mitochondrial matrix. CypD regulates opening of mitochondrial permeability transition pores. Both mechanisms may be affected by amyloid β peptides accumulated in mitochondria in Alzheimer's disease (AD). In order to clarify changes occurring in brain mitochondria, we evaluated interactions of both mitochondrial proteins in vitro (by surface plasmon resonance biosensor) and detected levels of various complexes of 17β-HSD10 formed in vivo (by sandwich ELISA) in brain mitochondria isolated from the transgenic animal model of AD (homozygous McGill-R-Thy1-APP rats) and in cerebrospinal fluid samples of AD patients. By surface plasmon resonance biosensor, we observed the interaction of 17β-HSD10 and cypD in a direct real-time manner and determined, for the first time, the kinetic parameters of the interaction (*k*_*a*_ 2.0 × 10^5^ M^1^s^−1^, *k*_*d*_ 5.8 × 10^4^ s^−1^, and *K*_*D*_ 3.5 × 10^–10^ M). In McGill-R-Thy1-APP rats compared to controls, levels of 17β-HSD10–cypD complexes were decreased and those of total amyloid β increased. Moreover, the levels of 17β-HSD10–cypD complexes were decreased in cerebrospinal fluid of individuals with AD (in mild cognitive impairment as well as dementia stages) or with Frontotemporal lobar degeneration (FTLD) compared to cognitively normal controls (the sensitivity of the complexes to AD dementia was 92.9%, that to FTLD 73.8%, the specificity to AD dementia equaled 91.7% in a comparison with the controls but only 26.2% with FTLD). Our results demonstrate the weakened ability of 17β-HSD10 to regulate cypD in the mitochondrial matrix probably via direct effects of amyloid β. Levels of 17β-HSD10–cypD complexes in cerebrospinal fluid seem to be the very sensitive indicator of mitochondrial dysfunction observed in neurodegeneration but unfortunately not specific to AD pathology. We do not recommend it as the new biomarker of AD.

## Introduction

The nucleus-encoded mitochondrial matrix protein 17β-hydroxysteroid dehydrogenase type 10 (17β-HSD10) is an essential protein operating via multiple enzymatic as well as non-enzymatic functions. Its deficiency, marked overexpression or loss of enzymatic function is associated with various pathologies [1–3]. Cytosolic 17β-HSD10 is imported into the mitochondrial matrix via phosphatase and tensin homologue-induced putative kinase 1 (PINK1)—mediated Parkin pathway [4, 5]. It has been suggested that direct interactions with Parkin protein play a key role in the regulation of levels of mitochondrial 17β-HSD10 and that Parkin overproduction increases 17β-HSD10 levels in the matrix [5]. Since Pink1-mediated Parkin pathway is involved also in the regulation of mitophagy and 17β-HSD10 overproduction protects mitochondria against their degradation, it appears that mitochondrial levels of 17β-HSD10 could be one of mechanisms by which Parkin preserves mitochondrial quality [5, 6].

17β-HSD10 is known as a binding partner of amyloid β (Aβ) peptides, accumulated in the brains of individuals with Alzheimer's disease (AD), which can lead among others to mitochondrial dysfunction [e.g., 7]. Experimental results revealed overexpression of 17β-HSD10 especially in cortical or hippocampal regions of AD patients when compared to age-matched controls [8, 9] which is also reflected by increased concentrations in cerebrospinal fluid (CSF) [10–12]. Mutual interactions of 17β-HSD10 and Aβ can be documented by many experiments in vitro [e.g., 2, 7, 9, 13–19], by their co-localization in brain mitochondria of AD patients or of transgenic (Tg) animal models of AD [9] and also by detection 17β-HSD10–Aβ complexes occurring in CSF [10, 11]. Experiments on Tg animals overexpressing 17β-HSD10 when compared to corresponding wild-type (WT) controls indicate its neuroprotective role against oxidative stress. However, double Tg animals overexpressing 17β-HSD10 and mutant human amyloid precursor protein display exaggerated AD-like pathology [7] and suggest that both overexpressed 17β-HSD10 and accumulated Aβ in mitochondria could play negative roles in mitochondrial dysfunction seen in AD [17]. With respect to the above-mentioned mutual interactions of 17β-HSD10 and Aβ, experimental results indicate that D loop of 17β-HSD10 (approximately 95 to 113 residues) and residues 12–24 of Aβ play important role here [2, 7, 9, 13, 15, 17–19]. Monomeric as well as oligomeric Aβ peptides can bind to 17β-HSD10 with K_D_ about 40–80 nM and the binding results in a distortion of 17β-HSD10 molecule including deformation of D loop and binding pocket for co-factor nicotinamide adenine dinucleotide [2, 7, 14, 15, 18]. It seems that oligomeric Aβ peptides, rather than monomeric Aβ, are able to significantly inhibit enzymatic activity of 17β-HSD10 [2, 7, 18].

It was hypothesised that 17β-HSD10 in the mitochondrial matrix binds to cyclophilin D (cypD) and that by preventing its translocation to the inner mitochondrial membrane it can regulate the opening of the mitochondrial permeability transition pore (MPTP) mediated by cypD [20]. Although interactions of 17β-HSD10 and cypD were predicted indirectly by co-immunoprecipitation and co-localization [20], the binding between 17β-HSD10 and cypD has not yet been confirmed experimentally. On the other hand, previous data found mutual interactions of cypD and Aβ in direct experiments in vitro using surface plasmon resonance (SPR) biosensor, and their co-localization in cortical mitochondria of AD patients and Tg animal models of AD [17, 21, 22]. It seems that oligomeric Aβ peptides have a higher affinity for binding to cypD than monomeric fragments [21]. Experimental results obtained on Tg animal model of AD suggest that the regulation of cypD by 17β-HSD10 could be influenced by Aβ accumulation in mitochondria and could lead to increased translocation of cypD from the matrix to the inner mitochondrial membrane [17].

The main aim of the present study was (i) to determine the kinetic parameters of the interaction between 17β-HSD10 and cypD in vitro using the SPR biosensor method and (ii) to estimate the levels of 17β-HSD10–cypD complexes formed in vivo either in brain mitochondria isolated from Tg rat model of AD or in CSF samples of AD patients in order to clarify changes occurring in brain mitochondria of individuals with AD in more detail and to evaluate CSF levels of 17β-HSD10–cypD complexes as a promising diagnostic biomarker of AD.

## Materials and Methods

### SPR Biosensor

#### Reagents

Sodium acetate (SA_10_, 10 mM, pH 5), 2-(N-morpholino)ethanesulfonic acid (MES, 10 mM, pH 5), 2-[4-(2-hydroxyethyl)piperazin-1-yl]ethanesulfonic acid (HEPES, 10 mM, pH 7.4), phosphate buffer (PBS, 1.4 mM KH_2_PO_4_, 8 mM Na_2_HPO_4_, 2.7 mM KCl and 137 mM NaCl, pH 7.4), NaCl, KCl, MgCl_2_, ethanolamine (EA) and bovine serum albumin (BSA) were purchased from Sigma-Aldrich, USA, in molecular biology grade or higher. Ethanol for spectroscopy (purity 99.9% or greater) was purchased from Merck, USA. Oligo-ethylene glycol (OEG) thiols terminated with carboxyl group (HS-C_11_-(EG)_6_-OCH_2_-COOH) and hydroxyl group (HS-C_11_-(EG)_4_-OH) were purchased from Prochimia, Poland. N-hydroxysuccinimide (NHS) and 1-ethyl-3-(3-dimethylaminopropyl)-carbodiimide hydrochloride (EDC) were purchased from GE Healthcare, USA. Human recombinant 17β-HSD10 and cypD proteins and monoclonal mouse antibody against mitochondrial cypD (anti-cypD) were purchased from Fitzgerald, USA. Aβ 142 fragment was obtained from AnaSpec, USA. All buffers were prepared using deionized water (Q-water, 18 MΩ/cm resistivity, Direct-Q UV3, Millipore, USA), phosphate buffer with high ionic strength (PBS_NaCl_) was prepared from PBS by increasing concentration of NaCl to 750 mM, HEPES_BSA_ was prepared from HEPES by addition of 250 µg/ml BSA, 15 mM KCl and 0.1 mM MgCl_2_.

#### Instrumentation

In this study, we used a laboratory 6-channel SPR platform based on the wavelength spectroscopy of surface plasmons (Plasmon VI) with dispersionless microfluidics [23, 24] developed at the Institute of Photonics and Electronics, Prague. In the SPR sensor, the angle of incidence of the light beam is fixed and the SPR dip is observed in the spectrum of polychromatic light coupled to a surface plasmon. The sensor response is expressed in terms of the shift in the wavelength at which the SPR dip occurs. This response is sensitive to changes in the refractive index caused by the binding of molecules to the surface of an SPR chip. A shift of 1 nm in the SPR wavelength represents a change in the protein surface coverage of 17 ng/cm^2^ [25]. SPR chips used in this work were prepared by coating microscope glass slides (Marienfeld, Germany) with thin layers of titanium (1–2 nm) and gold (48 nm) via e-beam evaporation in vacuum. All SPR experiments were performed at 25 °C and a flow rate of 20 µl/min.

#### Functionalization of an SPR Chip

The surface of SPR chips was functionalized with a self-assembled monolayer (SAM) of mixed carboxy-terminated and hydroxy-terminated OEG thiols, on which anti-cypD was immobilized using the amino-coupling as described previously [25]. Briefly, a 3:7 molar mixture of HS-C_11_-(EG)_6_-OCH_2_-COOH and HS-C_11_-(EG)_4_-OH thiols was dissolved in ethanol at a total concentration of 0.2 M. SPR chip was immersed in the thiol mixture for 10 min at 40 °C and stored for at least 12 h at room temperature. Then, the chip was rinsed with ethanol and Q-water and mounted into the SPR biosensor. The mixture of 12.5 mM NHS and 62.5 mM EDC (in Q-water) was injected for 10 min to the surface of SPR chip in order to activate carboxylic groups. Then, anti-cypD at a concentration of 5 µg/ml in SA_10_ was injected for 12 min to covalently attach anti-cypD to the activated carboxylic groups. The surface was consequently washed by PBS_NaCl_ (5 min) to remove the non-covalently bound molecules from a surface of SPR chip and by 0.5 M EA (5 min) to deactivate the remaining active esters.

#### Interaction Between cypD and 17β-HSD10

Interaction between cypD and 17β-HSD10 proteins was studied in the direct fashion using the detection and reference channels. Initially, MES was injected for 10 min into both channels functionalized with anti-cypD. Then, 2 µg/ml cypD was injected into the detection channel until the sensor response of 1.5 nm was achieved, while MES was continuously pumped through the reference channel. Then, MES (20 min) and PBS_NaCl_ (5 min) were injected into both channels, followed by HEPES_BSA_ until the stable baseline was obtained. Solutions of 17β-HSD10 at concentrations of 30, 50, 150, 300, and 500 nM in HEPES_BSA_ were prepared and incubated for 10 min at 37 °C to ensure the full tetramerization of 17β-HSD10 [26]. Then, 400 µl of HEPES_BSA_ was added to the solution to obtain the 17β-HSD10 at final concentrations of 6, 10, 30, 60, and 100 nM. These solutions were injected for 10 min into both detection (with attached cypD) and reference (without attached cypD) channels to monitor the association phase, followed by exposing the sensor surface to HEPES_BSA_ for 15 min to characterize the dissociation phase. The reference compensated curves (sensor responses from the reference channels were subtracted from those obtained in the particular detection channels) were globally fitted using BIAevaluation software version 4.1 and 1:1 Langmuir model considering mass transport effects. In order to provide the positive control for our biosensor experiments, we performed a control experiment in which we observed the binding of Aβ 1−42 to the cypD immobilized on the SPR sensor surface. In the immobilization step, 2 µg/ml cypD was injected into the detection channel until the sensor response leveled off (~ 5 nm), while MES was continuously pumped through the reference channel. Then, MES (20 min) and PBS_NaCl_ (5 min) were injected into both channels, followed by HEPES_BSA_ until the stable baseline was obtained. In the detection step, Aβ 1−42 (500 mM, incubated for 1 h at 37 °C) was injected into detection and reference channels and the binding was observed for 10 min.

### Co-immunoprecipitation Experiment to Detect Interaction of 17β-HSD10 and cypD

Mixtures of human full length recombinant 17β-HSD10 protein (His tag) (Fitzgerald Industries International) and of human full length cypD protein (His tag) (Fitzgerald Industries International) in PBS (concentration 20 µg/ml each; in 100 µl) were incubated for 2 h at room temperature and then mixed with 2 µl with either polyclonal rabbit anti-17β-HSD10 (Flarebio) or monoclonal mouse anti-cypD (Fitzgeral Industries International). Incubation was continued for another 2 h and mixtures were then transferred to tubes containing Protein A-sepharose (Sigma) pellet (approx. 50 µl) and incubated while rotating overnight at 4 °C. Samples were centrifuged (8000×*g* for 5 min), supernatants collected and the pellets were washed 3 times with PBS by centrifugation. Samples were then dissolved in Laemli denaturating sample buffer, electrophoresed in precast gradient 5–15% polyacrylamide Mini-Protean TGX gels and blotted to nitrocellulose in Trans-Blot Turbo according to Bio-Rad protocols. Blotted membranes were quenched by incubation in 10% soya milk in PBS, washed, incubated overnight at 4 °C in sealed pouch with either anti-17β-HSD10 or anti-cypD (dilution 1:1000 in both cases), washed and incubated with respective anti-immunoglobulin (IgG)-horseradish peroxidase (HRP) conjugate (either goat polyclonal anti-mouse IgG/HRP or goat polyclonal anti-rabbit IgG/HRP, dilutions 1:5000, both from Dako) for 2 h. To reveal immunoprecipitated proteins, blots were incubated in 20 ml PBS containing 10 mg of 3,3-diaminobenzidine (Sigma) plus 10 µl of 30% hydrogen peroxide and stained membranes were after washing imaged by ChemiDoc XRS + Imager (Bio-Rad).

### Animals and Isolation of Mitochondria

Ten 11-month old Tg homozygous male McGill-R-Thy1-APP rats and ten age-matched WT male Wistar controls (all from PsychoGenics, Austria) or 7-month old male Wistar rats (five control animals for preparation of internal standards, from Velaz, Czech Republic) were housed in cages (2 rats per cage) in a temperature-controlled room (21–22 °C), with a 12:12 h light/dark regime (lights on at 7:00 a.m.) with free access to food (ST-1 diet) and water. All manipulations were performed according to the Guidelines of the European Union Council (86/609/EU). Rats were sacrificed by cervical dislocation, decapitated, and the brains rapidly removed from the skulls. The left hemispheres (without the cerebellum, medulla oblongata and bulbus olfactorius) were dissected on an ice-cold plate, weighed and immediately used for isolation of mitochondria. Mitochondria were isolated by discontinuous Percoll density gradient [27]. Concentration of total mitochondrial proteins was estimated by means of Coomassie Brilliant Blue G-250 [28]. Mitochondria isolated from twenty 11-month old male rats (particular samples) and two 7-month old male rats (one mixed sample which aliquots are used as internal standards) were resuspended in the buffer (0.25 M sucrose, 0.5 mM K^+^-EDTA, 10 mM TRIS, pH 7.4) and adjusted to mitochondrial protein concentration of 9 mg/ml. Particular aliquots of mitochondria (250 µl) were stored at −40 °C until assayed.

### Human CSF Samples

All experiments were conducted in accordance with The Declaration of Helsinki. The study was approved by the Ethical Committees of Motol University Hospital, in accordance with the Laws 129/2003 and 130/2003 of the Czech Republic. Written informed consent was obtained from all study participants, either personally or by proxy. CSF samples were collected from 171 patients undergoing lumbar puncture as a part of their routine diagnostic work-up (basic characteristics are shown in Table 1). Patients were divided into five groups. The first group of 12 cognitively normal controls consisted of neurological patients with normal cognitive functions and normal basic CSF findings undergoing lumbar puncture for various medical reasons (e.g. facial palsy, headache) and of cognitively normal individuals undergoing elective orthopedic surgery in spinal anesthesia who agreed to give 6 ml of CSF for research purposes during the procedure. Only subjects with normal levels of Aβ 1–42, total-τ and phospho-τ and with normal basic analysis of CSF were included in the control group. The second group consisted of 17 patients with mild cognitive impairment not related to AD (MCI-others) and the third group of 44 patients fulfilled the criteria for mild cognitive impairment due to AD (MCI-AD) [29]. The fourth group consisted of 56 patients with probable AD dementia (ADD) [30]. And finally the fifth group comprised 42 patients with Frontotemporal lobar degeneration (FTLD) [31]. All patients underwent complex diagnostic process including neuropsychological examination by neuropsychological battery (including the Mini Mental State Examination test), brain MRI, neurological examination and routine blood tests. All CSF samples were obtained by lumbar puncture with an atraumatic needle in the lying position. The first 3 ml was used for routine analysis (cell count, total protein etc.), and the remaining sample was centrifuged and frozen in polypropylene tubes at − 80 °C 30 min after the puncture, until required for further analyses. Particular aliquots of mixed CSF samples from eight people discarded from the study (four men and four women) were used as internal standards.

**Table 1:** Characteristics of patients providing CSF samples

Groups	n	Sex (M/F)	Age (years)	MMSE score
Controls	12	6/6	67.6 ± 6.6	28.5 ± 0.9
MCI-others	17	11/6	68.7 ± 9.9	27.2 ± 1.6
MCI-AD	44	21/23	72.2 ± 6.7	25.5 ± 1.8***
ADD	56	18/38	71.0 ± 7.9	20.6 ± 3.7***
FTLD	42	17/25	64.9 ± 7.5	21.0 ± 6.2***
Totally: Welch test	171	73/98	p < 0.001	p < 0.001

Data are presented as the mean ± SD

Statistical significance (Bonferroni adjusted t-test, separate variance) was calculated with respect to controls (*** adjusted p < 0.001). Group of ADD patients was significantly older when compared to FTLD people.

*M* males, *F* females, *MMSE* mini-mental state examination, *MCI-others* individuals with mild cognitive impairment not related to Alzheimer's disease, *MCI-AD* mild cognitive impairment due to Alzheimer's disease, *ADD* Alzheimer's disease dementia, *FTLD* Frontotemporal lobar degeneration

### Enzyme-Linked Immunosorbent Assay (ELISA)

#### Quantitative Competitive ELISA for Estimation of 17β-HSD10 in Rat Brain Mitochondrial Fraction

A full length rat recombinant protein 17β-HSD10 (Flarebio) was dissolved in carbonate buffer (75 mM NaHCO_3_, 25 mM Na_2_CO_3_, pH 9.5). The solution was applied to 96-well polystyrene plates (Nunc Immuno Plate Maxisorp, 86 ng of protein per well) and incubated for 4 h at room temperature (shaker IKA, MTS 2/4). Plates were subsequently washed three times with PBS (2.8 mM Na_2_HPO_4_, 7.2 mM NaH_2_PO_4_, 100 mM NaCl, pH 7.2). Nonspecific binding was suppressed by incubation with blocking solution (10 mg/ml of bovine serum albumin in carbonate buffer) for 1 h at room temperature followed by overnight incubation at 10 ºC. Prior to the assay, the plates were washed three times with washing solution (PBS and 0.05% Tween 20). A standard curve was prepared using 138 to 4839 ng of rat recombinant protein 17β-HSD10 dissolved in 10% dimethyl sulfoxide. Various volumes of standards, 50 µl of particular samples (100 µl of mitochondrial fraction and 20 µl of redistilled water and 10 µl of 15% n-dodecyl β-d-maltoside) and 50 µl of internal standards (prepared in accordance with the samples) were pipetted in duplicates and adjusted to 100 µl by 10% dimethyl sulfoxide. Plates were subsequently incubated with primary antibody (polyclonal rabbit anti-17β-HSD10 reacting with human, mouse, rat and monkey protein, Flarebio, dilution 1:4000) for 4 h at room temperature. Plates were washed four times with washing solution (PBS with 0.05% Tween 20), and secondary antibody (polyclonal swine anti-rabbit IgG/HRP (DakoCytomation, dilution 1:6000) was added. After a 1-h incubation at room temperature, the plates were washed four times with washing solution. Subsequently, substrate solution (3, 3′,5 ,5′-tetramethylbenzidine liquid substrate system for ELISA, Sigma) was added and plates were incubated for 20 min at room temperature. The reaction was stopped with 0.5 M H_2_SO_4_ and plates were measured at the wavelength of 450 nm using an automated plate reader (Multiskan EX, Thermo). After the subtraction of the blank, levels of 17β-HSD10 were estimated in particular samples. The detection limit was determined to be about 20 ng of the protein. Intra- and inter-assay variability was determined to be 3.7% and 9.6% at the control protein level of 277 ng.

#### Quantitative Sandwich ELISA for Estimation of cypD in Rat Mitochondrial Fraction

Levels of rat cypD were estimated by a kit (MyBioSource). 50 µl of samples (250 µl of mitochondrial fraction and 25 µl of 15% n-dodecyl β-d-maltoside) were incubated in quadruplicates and measured in accordance with manufacturer’s instructions.

#### Semi-quantitative Sandwich ELISA for Estimation of Total Aβ in Rat Mitochondrial Fraction

Plate was coated by goat anti-mouse IgG (Fitzgerald Industries International, 1 µg per well) and then by monoclonal mouse anti-Aβ 13–28 recognizing mouse, rat and human Aβ (Sigma, 100 ng per well). 100 µl of samples or internal standards (225 µl of mitochondrial fraction and 45 µl of 15% n-dodecyl β-d-maltoside in both cases) were incubated in duplicates for 5 h. Subsequently, second primary antibody (polyclonal rabbit anti-Aβ 1–14 reacting with mouse, rat and human fragments, Abcam, dilution 1:9000) and lately secondary antibody (swine anti-rabbit IgG/HRP, DakoCytomation, dilution 1:6000) were applied. After the subtraction of the blank, the values were compared to those from the internal standards and expressed in percentage.

#### Semi-quantitative Sandwich ELISA for Estimation of 17β-HSD10–cypD or 17β-HSD10–Total Aβ Complexes in Rat Mitochondrial Fraction

Two plates were coated by a capture antibody against 17β-HSD10 (polyclonal rabbit anti-ERAB reacting with human, mouse, rat and monkey protein, Flarebio) dissolved in carbonate buffer. Coating solution (246 ng of antibody per well) was applied and plates were incubated for 5 h at room temperature. 100 µl of samples or internal standards (250 µl of mitochondrial fraction and 150 µl of redistilled water and 25 µl of 15% n-dodecyl β-d-maltoside) were incubated in duplicates for 5 h. Subsequently, second primary antibody against cypD (monoclonal mouse anti-PPIF recognizing rat and human protein, Abnova, dilution 1:2133) and lately secondary antibody (polyclonal rabbit anti-mouse IgG/HRP, Dako, dilution 1:6000) were applied on the first plate. Analogously, second primary antibody against total Aβ (monoclonal mouse anti-Aβ 13–28 recognizing mouse, rat and human fragments, Sigma, dilution 1:2666) and lately secondary antibody (polyclonal rabbit anti-mouse IgG/HRP, Dako, dilution 1:6000) were added to the second plate. After the subtraction of the blank, the values were compared to those from the internal standards and expressed in percentage.

#### Quantitative Sandwich ELISA for Estimations of Levels of Aβ 1–42, τ and Phospho-τ in Human CSF

Levels of human Aβ 1–42, total τ and τ phosphorylated at position 181 were estimated by three kits (Fujirebio) according to the manufacturer’s instructions.

#### Semi-quantitative Sandwich ELISA for Estimation of 17β-HSD10–cypD Complexes in Human CSF

Plate was coated by goat anti-rabbit IgG (Fitzgerald Industries International, 1 µg per well) and then by polyclonal rabbit anti-17β-HSD10 (polyclonal rabbit anti-ERAB recognizing human, mouse, rat and monkey protein, Flarebio, 200 ng per well). 100 µl of redistilled water or of CSF samples were incubated in duplicates for 4 h. Subsequently, second primary antibody against cypD (monoclonal mouse anti-PPIF recognizing mouse, rat and human protein, Abnova, dilution 1:2133) and lately secondary antibody (donkey anti-mouse IgG/HRP, Dako, dilution 1:8000). After the subtraction of the blank, the values were compared to those from the internal standards (mixed CSF samples of totally eight people discarded from the study) and expressed in percentage.

### Transmission Electron Microscopy (TEM)

Sample of mitochondria was stored in 0.25 M sucrose, 0.5 mM K^+^-EDTA, 10 mM Tris (pH 7.4) with 10% dimethyl sulfoxide in protein concentration of 5 mg/ml at − 80 °C. Before TEM analysis, the sample was tempered to room temperature and centrifuged at 14,000×*g* for 10 min. The pellet was fixed with 2.5% glutaraldehyde in 0.1 M cacodyladate buffer with 5 mM CaCl_2_ and embedded in 2% agar. The second fixation was done with 1% osmium tetroxide in 0.1 M cacodyladate buffer, serial dehydration and embedding into epoxy resin. The images were acquired with FEI Morgagni 268 transmission electron microscope operated at 80 kV equipped with Mega View III CCD camera (pixel size 6.45 × 6.45 µm).

### Statistical Analysis

Statistical analysis was performed using BMDP statistical software. Parametric Welch test for global and Bonferroni adjusted t-test (separate variance) for pairwise comparisons (program 7D) were used (*Adjusted p < 0.050, **Adjusted p < 0.010, ***Adjusted p < 0.001). In addition, correlation analysis (program 6D) was applied. The equality of correlation coefficients (Rs) in two groups was examined using the test based on Fisher´s Z-transformation (Z test, two-tailed version) [32]. Sensitivity and specificity using Receiver Operating Characteristic (ROC) curve analysis were calculated in MS Excel in accordance with MedCalc statistical software manual. Data are presented as means ± S.D.

## Results

### SPR Biosensor

**Fig. 1:**
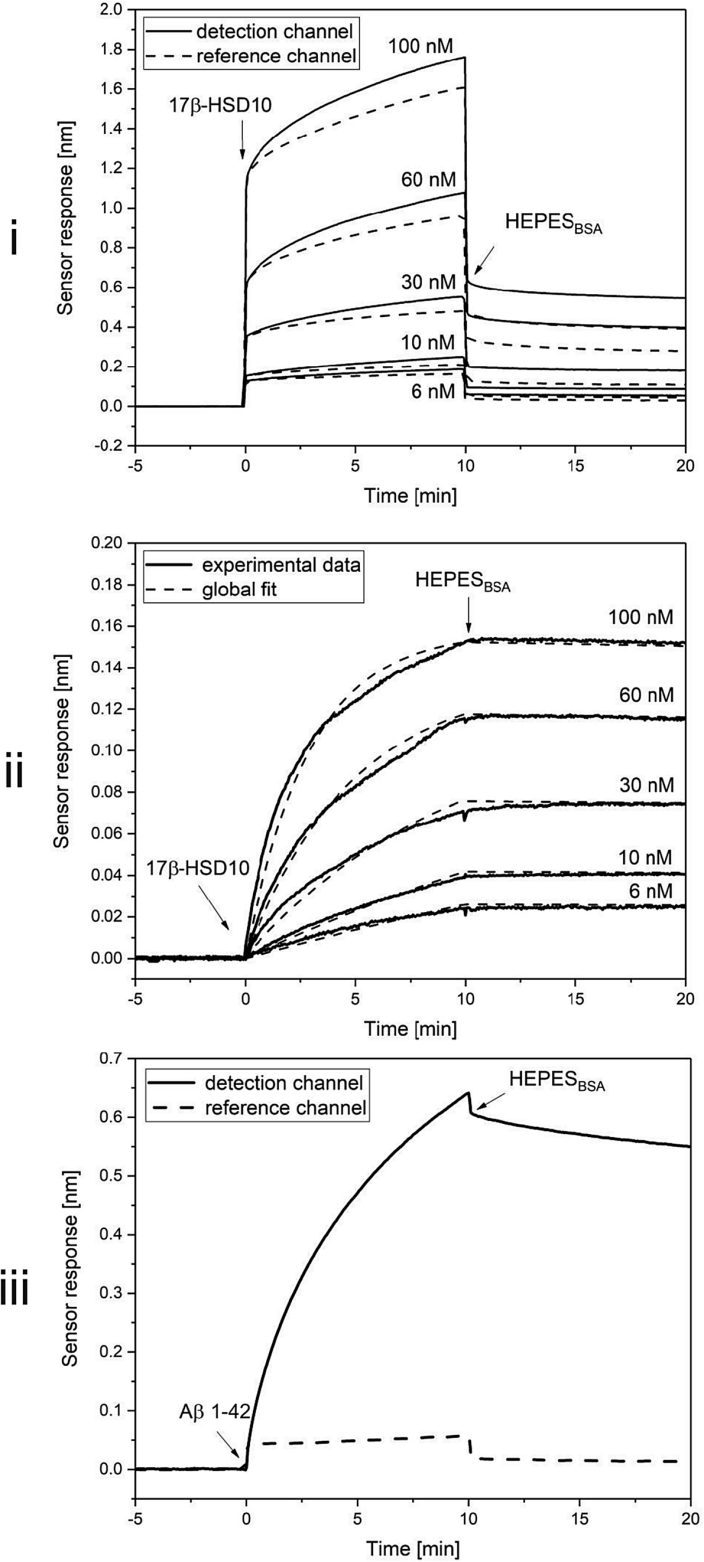
Data of SPR biosensor demonstrating the binding of 17β-HSD10 to cypD. Figure demonstrates: (i) the sensor response to binding of 6, 10, 30, 60, and 100 nM 17β-HSD10 in the detection (solid lines) and in the reference (dashed lines) channels, (ii) reference-compensated binding curves for the set of the used concentrations of 17β-HSD10 (solid lines) and their global fits (dashed lines) and finally (iii) the sensor response to the binding of Aβ 1-42 in the detection (solid line) and in the reference (dashed line) channel

We used the SPR biosensor to demonstrate that mitochondrial proteins cypD and 17β-HSD10 interact with each other and to determine kinetic parameters of the interaction. In the used detection format, we injected different concentrations of 17β-HSD10 into both the detection (immobilized cypD) and reference (absence of cypD) channels. Figure 1i shows significantly higher sensor response in the detection channel than in the reference channel, which indicates specific interaction between 17β-HSD10 and cypD. The data obtained in the reference channel were subtracted from those obtained in the detection channel to compensate for bulk refractive index changes and non-specific binding to the surface. The reference-compensated binding curves obtained for the set of five different concentrations of 17β-HSD10 were further used to determine the kinetic parameters of the interaction between 17β-HSD10 and cypD (Fig. 1ii). The association rate constant *k*_*a*_, dissociation rate constant *k*_*d*_, and dissociation equilibrium constant *K*_*d*_ were determined as 2.0 × 10^5^ M^1^s^−1^, 5.8 × 10^4^ s^−1^, and 3.5 × 10^−10^ M, respectively. In order to confirm that the immobilized cypD is capable of interacting with its binding partners, we used Aβ 1-42 as a positive control (Aβ 1-42 is known to specifically interact with cypD [21]). The corresponding sensorgram is shown in the Fig. 1iii and proves that cypD can indeed interact with Aβ 1-42.

#### Co-immunoprecipitation

**Fig. 2:**
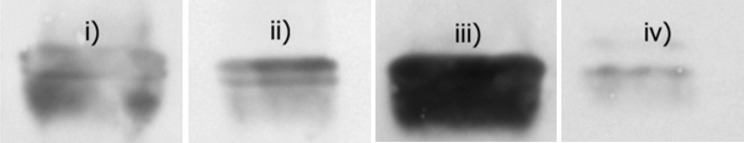
Demonstration of 17β-HSD10–cypD interaction by co-immunoprecipitation. Figure demonstrates: (i) immunoblot of 17β-HSD10 standard (cca 1 µg), (ii) co-immunoprecipitation of 17β-HSD10–cypD mix pulled-down by anti-cypD antibody-Protein A-sepharose and revealed by anti-17β-HSD10 antibody, (iii) immunoblot of cypD standard (cca 2 µg), and finally (iv) co-immunoprecipitation of 17βHSD10–cypD mix pulled-down by anti-17β-HSD10 antibody-Protein A-sepharose and revealed by anti-cypD antibody

We used also co-immunoprecipitation experiment to demonstrate the interaction of mitochondrial proteins 17β-HSD10 and cypD. Figure 2 supports notion of such binding (blot ii, blot iv).

#### Experiments Performed on Rat Brain Mitochondrial Fraction

**Fig. 3:**
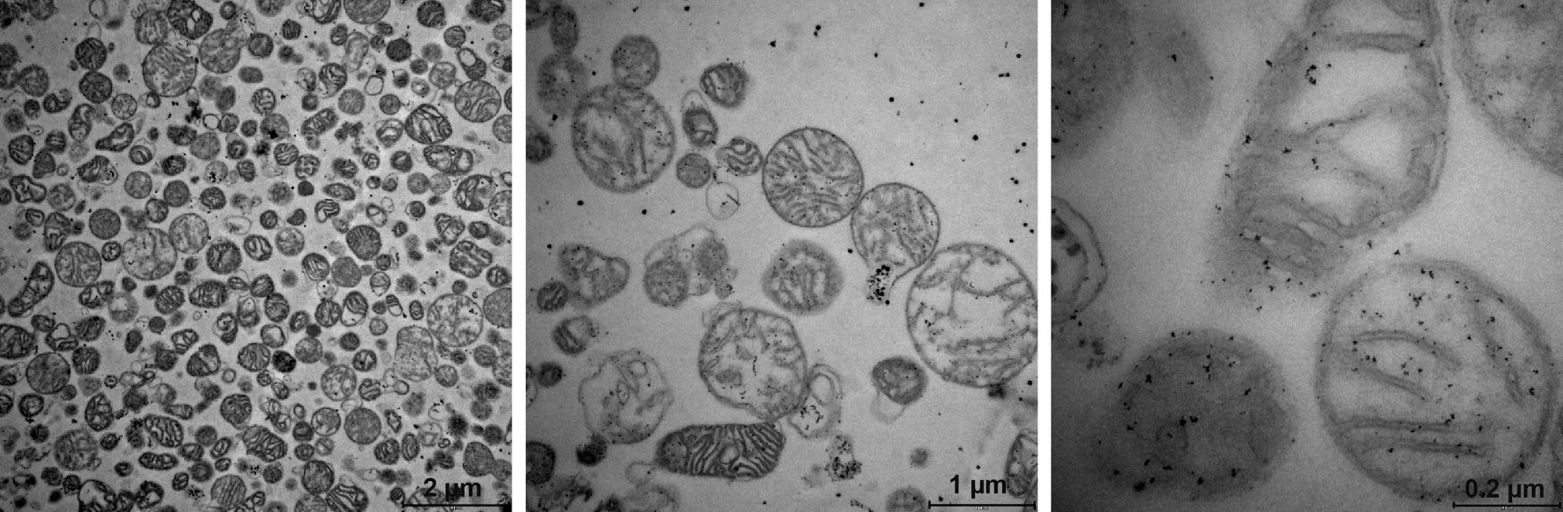
Pictures of isolated mitochondria by transmission electron microscopy. Transmission electron microscopy (Imaging Methods Core Facility, BIOCEV, Prague) documented high purity and intactness of mitochondria isolated in accordance with the study of Rajapakse et al. [27]. Sample (mitochondria isolated from three 7-month old male Wistar rats used as internal standards) was stored in 0.25 M sucrose, 0.5 mM K^+^-EDTA, 10 mM Tris, pH 7.4 with 10% dimethyl sulfoxide at − 80 °C (in concentration of 5 mg protein/ml).

TEM documented high purity and intactness of our isolated mitochondria (Fig. 3). Intactness of mitochondria was also supported by enzymatic assays (data not shown). In particular, activities of citrate synthase (a marker of the mitochondrial matrix) and of lactate dehydrogenase (a cytosolic marker) were estimated by kits of Sigma-Aldrich. Citrate synthase assay revealed that 91% of mitochondria remained their inner membrane intact. Lactate dehydrogenase assay indicated that activity of a cytosolic enzyme was approximately 40-times lower in fraction of purified mitochondria (37 mU/ml) than in original homogenate (1381 mU/ml).

**Fig. 4:**
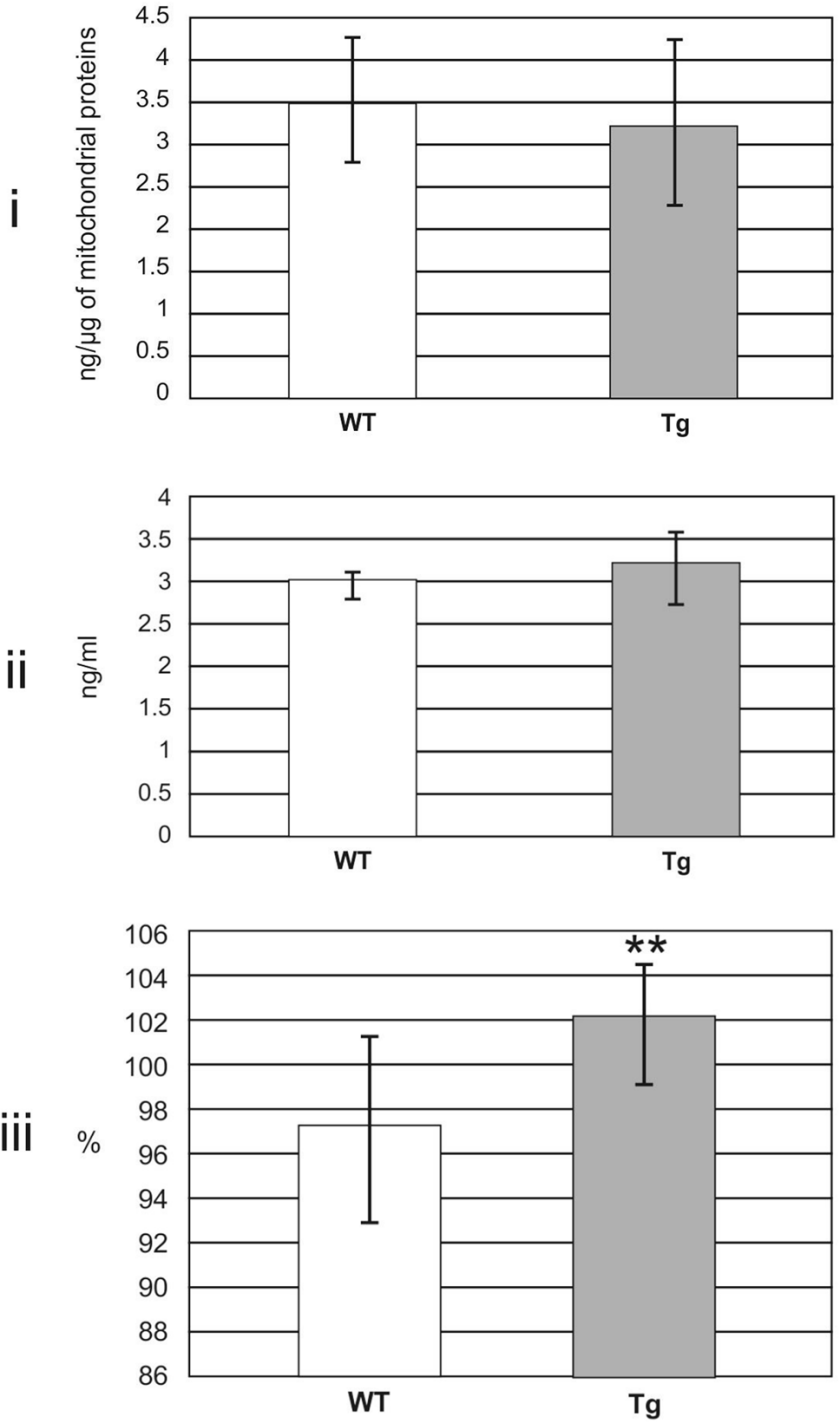
Levels of 17β-HSD10, cypD and Aβ in rat mitochondrial fraction estimated by ELISA. Data are presented as the mean ± SD. All values were obtained from 10 WT and 10 Tg (homozygous McGill-R-Thy1-APP) rats. Figure demonstrates: (i) levels of 17β-hydroxysteroid dehydrogenase type 10 (17β-HSD10) estimated by quantitative competitive ELISA (differences between both groups were not statistically significant, Welch test: p = 0.508), (ii) levels of cyclophilin D (cypD) estimated by quantitative sandwich ELISA (differences between both groups were not statistically significant, Welch test: p = 0.091), and finally (iii) total levels of amyloid β (Aβ) peptides estimated by semi-quantitative ELISA (differences between both groups were statistically significant, Welch test: p = 0.006)

**Fig. 5:**
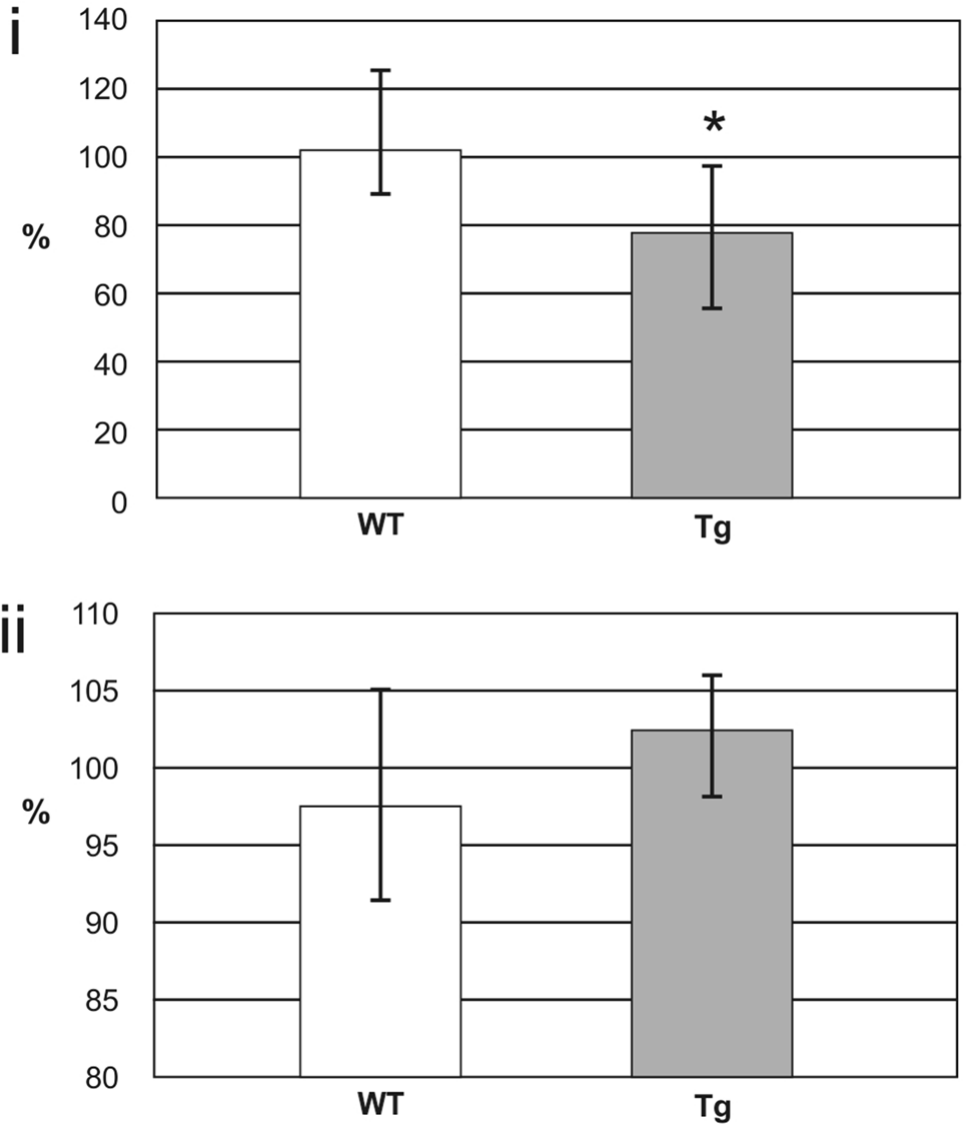
Levels of 17β-HSD10–cypD and 17β-HSD10–Aβ complexes in rat mitochondrial fraction estimated by semi-quantitative sandwich ELISA. Data are presented as the mean ± SD. Levels of two types of complexes, i.e. of 17β-hydroxysteroid dehydrogenase type 10 and cyclophilin D (17β-HSD10–cypD complexes, experiment (i) and of 17β-hydroxysteroid dehydrogenase type 10 and amyloid β (17β-HSD10–Aβ complexes, experiment (ii) were obtained from 10 WT and 10 Tg (homozygous McGill-R-Thy1-APP) rats. The values of 17β-HSD10–cypD complexes were significantly decreased in Tg compared to WT rats (Welch test: p = 0.012). On the contrary, no differences were found in levels of 17β-HSD10–Aβ complexes (Welch test: p = 0.072)

Results of quantitative estimations are seen in Figs. 4i and ii. Figure 4i shows levels of 17β-HSD10 estimated by competitive ELISA. Values of WT rats equaled 3.5 ± 0.7 ng/µg of mitochondrial proteins, differences between Tg and WT groups were not statistically significant (Welch test: p = 0.508). Figure 4ii demonstrates levels of cypD estimated by sandwich ELISA. The values of WT rats equaled 3.0 ± 0.1 ng/ml and differences between both groups were not statistically significant again (Welch test: p = 0.091). Results of semi-quantitative ELISA experiments are demonstrated in Figs. 4iii and 5. All values were compared to those from the internal standards and expressed in percentage. Figure 4iii shows total levels of mitochondrial Aβ estimated by a sandwich ELISA. Results indicated the significant increase to 105.0% in Tg when compared to WT rats (Welch test: p = 0.006). Figure 5 demonstrates levels of 17β-HSD10–cypD and 17β-HSD10–Aβ complexes estimated by sandwich ELISA. While the values of 17β-HSD10–cypD complexes were significantly decreased in Tg compared to WT rats (to 76.2%, p = 0.012), no significant differences were found in the case of 17β-HSD10–Aβ complexes (p = 0.072).

Results of correlation analysis performed on data obtained from rat mitochondrial fraction revealed two significant negative correlations in WT control rats—the first between 17β-HSD10 and cypD levels (R = − 0.708, p = 0.022) and the second between 17β-HSD10–cypD and 17β-HSD10–Aβ complexes (R = − 0.745, p = 0.013). The correlation between 17β-HSD10 and cypD levels was not significant in Tg rats (R = + 0.316, p = 0.374) and the result of Z–test supported the significant change between Tg and WT rats (p = 0.024). Analogously, the correlation between 17β-HSD10–cypD and 17β-HSD10–Aβ complexes was not significant in Tg animals (R = − 0.071, p = 0.845), however, the result of Z-test indicated only borderline significance (p = 0.095).

#### Experiments Performed on Human CSF Samples

**Fig. 6:**
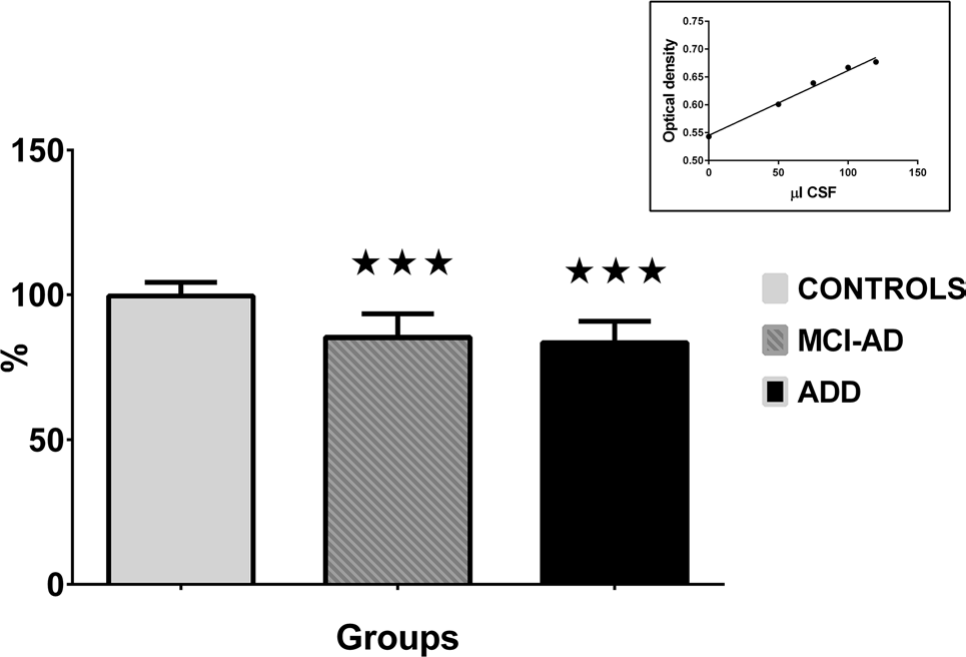
Levels of 17β-HSD10–cypD complexes in CSF of people with AD estimated by semi-quantitative sandwich ELISA. Data (a part of results of Table 2) are presented as the mean ± SD. Levels of complexes were estimated in CSF samples of controls (n = 12), of people with mild cognitive impairment due to Alzheimer's disease (MCI-AD, n = 44) and of people with Alzheimer's disease dementia (ADD, n = 56). Statistical significance (Bonferroni adjusted t-test, separate variance) was calculated with respect to controls (***Adjusted p < 0.001)

Analysis of CSF samples (Table 2) revealed significant changes in levels of Aβ 1–42 in all evaluated groups (the drops to 70.4% in MCI-others, to 53.6% in MCI-AD, to 49.5% in ADD, and to 68.1% in FTLD) when compared to controls. Significant changes in levels of total-τ (the increases to 191.9% in MCI-AD, to 221.8% in ADD and to 174.2% in FTLD) were also observed. In levels of phospho-τ, the increase to 139.2% in ADD was found compared to FTLD group. And finally, levels of 17β-HSD10–cypD complexes were significantly decreased (the drops to 85.6% in MCI-AD, to 83.9% in ADD and to 89.9% in FTLD) when compared to controls (a part of the results is presented also in Fig. 6, in addition, the insert in the top-right corner shows dependency of optical density on CSF volume). Positive levels of 17β-HSD10–cypD complexes were defined as values below the cut-off (< 93.0%). The sensitivity of the biomarker to ADD was 92.9% (to MCI-AD was 86.4% and to FTLD was 73.8%). A comparison with non-demented controls revealed the specificity of 91.7% in ADD group, however, that with FTLD only of 26.2%.

**Table 2:** Results of CSF analysis

Groups	Aβ 1–42 (pg/ml)	τ (pg/ml)	Phospho-τ (pg/ml)	17β-HSD10–cypD (%)
Controls	986.3 ± 225.3	278.7 ± 124.9	50.2 ± 23.0	99.6 ± 4.7
MCI-others	694.5 ± 221.7*	354.2 ± 225.5	52.2 ± 26.3	96.2 ± 3.9
MCI-AD	528.2 ± 196.4***	534.8 ± 355.0**	76.8 ± 52.8	85.3 ± 8.1***
ADD	488.1 ± 166.7***	618.2 ± 446.0***	71.0 ± 35.5	83.6 ± 7.2***
FTLD	672.1 ± 237.9**	485.5 ± 320.3*	51.0 ± 23.3	89.5 ± 7.3***
Welch test	p < 0.001	p < 0.001	p = 0.003	p < 0.001

Data are presented as the mean ± SD

CSF samples were obtained from patients of Table 1. The levels of 17β-HSD10–cypD complexes were compared to those from the internal standards (mixed CSF samples of eight people discarded from the study) and expressed in percentage. Statistical significance (Bonferroni adjusted t-test, separate variance) was calculated with respect to controls (*Adjusted p < 0.050, **Adjusted p < 0.010, ***Adjusted p < 0.001). The levels of phospho-τ were significantly increased in ADD patients compared to FTLD people.

Results of correlation analysis performed on data from CSF samples revealed one significant negative correlation between age and Aβ 1–42 (R = − 0.693, p = 0.012) and one significant positive correlation between total-τ and phospho-τ levels (R = + 0.970, p < 0.001) in the control group. The results of Z-test indicated a significant correlation loss in ADD (age vs Aβ 1–42: R = − 0.034, p = 0.803, Z-test: p = 0.023; τ vs phospho-τ: R = + 0.822, p < 0.001, Z-test: p = 0.010) and FTLD groups (age vs Aβ 1–42: R = − 0.106, p = 0.505, Z-test: p = 0.043; τ vs phospho-τ: R = + 0.688, p < 0.001, Z-test: p < 0.001) compared to the controls.

## Discussion

In this work, we investigated the interaction between 17β-HSD10 and cypD in vitro using the SPR biosensor method and, for the first time, determined the kinetic parameters of this interaction. The interaction analysis revealed that 17β-HSD10 and cypD interact with high affinity and form a stable complex (Fig. 1i–ii). This result can be supported also by co-immunoprecipitation experiment (Fig. 2, blots ii and iv). Moreover, we used ELISA to detect 17β-HSD10–cypD complexes formed in vivo in rat brain mitochondria (Fig. 5i) and in human CSF (Table 2, Fig. 6). However, before discussing this interaction, it is worthwhile to look at the interacting biomolecules separately.

With respect to particular levels of 17β-HSD10 and cypD, we have to note that our data obtained from quantitative ELISA did not reflect real concentrations occurring in the brain mitochondrial matrix of 11-month old WT rats since mitochondria were previously concentrated during the isolation process. However, the ratio of their size could be determined after recalculation of data to original mitochondrial fraction. Since the concentration of functional 17β-HSD10 homotetramers was around 0.135 µM (MW of human protein = 26.9 kDa) and that of cypD equaled 0.017 µM (MW of human protein = 18.9 kDA), it seems that the levels of tetrameric 17β-HSD10 are more than 7 times higher in the mitochondrial matrix when compared to those of cypD. Incidentally, it is in line with the fact that 17β-HSD10 is the essential protein with enzymatic as well as non-enzymatic functions necessary for structural and functional integrity of mitochondria [3]. While mutational inactivation of 17β-HSD10 results in lethal phenotype [9], homozygous mice missing mitochondrial cypD survive but are more anxious and better in learning/memory tasks [22, 33]. It is important to point out that our results of correlation analysis revealed two significant negative correlations in WT rats (either between 17β-HSD10 and cypD levels or between 17β-HSD10–cypD and 17β-HSD10–Aβ complexes) which can be interpreted by a possible homeostatic interplay between levels of both mitochondrial proteins and by a potential competition for binding of cypD or Aβ to 17β-HSD10 in the matrix.

With respect to AD, the strength of the study is a well chosen animal model representing the human pathology. McGill-R-Thy1-APP rat model of AD is based on expression of human Aβ precursor protein carrying both the Swedish and Indiana mutations displays deficits in cognitive functions already in 3-month old rats [34] and cognitive changes are still more prominent in older animals [34–36]. Rats are characterized by intraneuronal accumulation of Aβ already in 1-week old rats [34] and by extracellular Aβ deposits from the age of 6 months [34, 36]. Moreover, experimental results indicate that 6-month old McGill-R-Thy1-APP rats and older ones display perturbations in neuronal mitochondria [37, 38] which well agree with increased total Aβ in brain mitochondrial fraction in this study (Fig. 4iii).

Our experiments performed on the rat brain mitochondrial fraction isolated from the whole left hemisphere did not reveal significant differences between Tg and WT rats in 17β-HSD10 (Fig. 4i) or cypD levels (Fig. 4ii). The results do not contradict findings of marked overexpressions of both mitochondrial proteins exclusively in extremely vulnerable brain regions (as the cortex or hippocampus) of individuals with AD or of Tg rodent models of AD [2, 7–9, 17, 22]. On the other hand, the enhanced concentrations of 17β-HSD10 in CSF of individuals with AD [10–12] suggest that the moderate average increase in 17β-HSDS10 expression in the brains of Tg models of AD should be expected. We assume that up-regulation and overexpression of 17β-HSD10 in the extremely vulnerable brain regions of McGill-R-Thy1-APP rats is very probable but its reduced level in the mitochondrial matrix may be due to another mechanism. E.g., it has been demonstrated that Aβ accumulated in both outer and inner mitochondrial membranes can block transports of nucleus-encoded mitochondrial proteins [39]. It seems therefore that Aβ-related blocking of Pink1-mediated Parkin pathway could also partly eliminate levels of originally up-regulated 17β-HSD10 (and perhaps also these of cypD) in the mitochondrial matrix. Our experiments support it via increased levels of mitochondrial Aβ (Fig. 4iii, note that it is not possible to distinguish between mitochondrial membrane-bound Aβ and free/bound Aβ in the matrix in our experiments) and perhaps also via missing significant changes in 17β-HSD10–Aβ complexes (Fig. 5ii). However, although the alterations are not significant here, a trend to the increase of 17β-HSD10–Aβ complexes is evident (see also the borderline significance value).

On the other hand, despite of unchanged levels of 17β-HSD10 (Fig. 4i) and cypD (Fig. 4ii) in mitochondria of McGill-R-Thy1-APP rats, 17β-HSD10–cypD complexes were significantly decreased (Fig. 5i). In addition, we observed the significant loss of negative correlation between 17β-HSD10 and cypD levels or the similar change with borderline significance in correlations between 17β-HSD10–cypD and 17β-HSD10–Aβ complexes (Results). The result obtained on Tg animal model of AD can be also supported by our results on human CSF samples from AD patients (Table 2, Fig. 6). This can be interpreted via Aβ-related dysbalance in levels of mitochondrial 17β-HSD10 and cypD and via trend to dysbalance among various complexes of 17β-HSD10 which can finally lead to the weakened function of 17β-HSD10 to regulate cypD in the matrix, in accordance with literature [20]. It seems that especially some fragments of accumulated Aβ via its enhanced binding to 17β-HSD10 could eliminate the interaction of 17β-HSD10 and cypD [40] and thus decrease the levels of 17β-HSD10–cypD complexes in the matrix. Free cypD could consequently translocate to the inner mitochondrial membrane and open MPTP. It is well known that MPTP opening results in inner membrane potential collapse, respiratory chain uncoupling, halt of mitochondrial adenosine triphosphate synthesis, and eventually mitochondrial swelling, rupture, and cell death [20]. We thus suggest that the weakened function of 17β-HSD10 to regulate cypD in the mitochondrial matrix can be directly involved in mitochondrial dysfunction.

Incidentally, although levels of 17β-HSD10–Aβ complexes are not significantly increased in mitochondria of McGill-R-Thy1-APP rats (Fig. 5ii), the balance between 17β-HSD10–cypD and 17β-HSD10–Aβ complexes seems to be moderately deflected in AD after all towards 17β-HSD10 bounded to Aβ. Our new results of SPR biosensor indicate that dual 17β-HSD10–cypD and 17β-HSD10–Aβ as well as tertial 17β-HSD10–Aβ–cypD complexes can be created in vitro and that there are pronounced effects of ionic composition/pH and particular Aβ fragments on the interaction of 17β-HSD10 and cypD [40]. We suppose that missing significant changes in 17β-HSD10–Aβ complexes in mitochondria of McGill-R-Thy1-APP rats may be based not only on unchanged 17β-HSD10 but also on the above-mentioned tertial complexes probably undetected by our sandwich ELISA.

And finally, our results revealed that CSF levels of 17β-HSD10–cypD complexes can be a very sensitive biomarker of AD including early disease stages (compare the very high sensitivity to MCI-AD 86.4% with that to ADD 92.9%). However, its specificity to AD pathology remains questionable when taking into account the similar results in FTLD patients. It seems that CSF levels of 17β-HSD10–cypD complexes reflect mitochondrial dysfunction in agreement with previous findings of mitochondrial dysfunction as a common feature of neurodegeneration [41]. On the other hand, 17β-HSD10–cypD complexes in CSF, similarly as Aβ–τ complexes evaluated in our older study [42], do not reflect degree of cognitive impairment (we did not observe significant correlations with MMSE in ADD (R = − 0.035, p = 0.801) as well as in FTLD (R = + 0.098, p = 0.536) in this study). Taken together, the advantages of this new biomarker (its high sensitivity to early as well as later stages of AD) are not greater than the disadvantages (its less specificity, the levels do not markedly reflect disease progression or degree of cognitive impairment) and thus we do not recommend it as the new biomarker of AD.

## Conclusions

In this work, we used the SPR biosensor method to observe in vitro interaction of the two nucleus-encoded mitochondrial proteins, 17β-HSD10 and cypD, and, for the first time, determined kinetic parameters of the interaction. The interaction analysis revealed that the two proteins exhibit high affinity towards each other and form a stable complex. In brain mitochondria isolated from Tg animal model of AD (McGill-R-Thy1-APP rats) and in CSF samples of AD patients, we observed the significant drops in levels of 17β-HSD10–cypD complexes which could indicate the weakened function of 17β-HSD10 to regulate cypD in the mitochondrial matrix. CSF levels of 17β-HSD10–cypD complexes seem to be the very sensitive indicator of mitochondrial dysfunction observed in neurodegeneration but unfortunately not specific to AD pathology. We do not recommend it as the new biomarker of AD.

## Abbreviations

17β-HSD10: 17β-hydroxysteroid dehydrogenase type 10
PINK1: Phosphatase and tensin homologue-induced putative kinase 1
Aβ: Amyloid β
AD: Alzheimer's disease
CSF: Cerebrospinal fluid
Tg: Transgenic
WT: Wild type
cypD: Cyclophilin D
MPTP: Mitochondrial permeability transition pore
SPR: Surface plasmon resonance
SA: Sodium acetate
MES: 2-(*N*-morpholino)ethanesulfonic acid
HEPES: 2-[4-(2-Hydroxyethyl)piperazin-1-yl]ethanesulfonic acid
PBS: Phosphate buffer
EA: Ethanolamine
BSA: Bovine serum albumin
OEG: Oligo-ethylene glycol
NHS: N-hydroxysuccinimide
EDC: 1-Ethyl-3-(3-dimethylaminopropyl)-carbodiimide hydro-chloride
Q-water: Deionised water
SAM: Self-assembled monolayer
ELISA: Enzyme-linked immunosorbent assay
IgG: Immunoglobulin
HRP: Horseradish peroxidase
MCI-others: Mild cognitive impairment not related to AD
MCI-AD: Mild cognitive impairment related to AD
ADD: Alzheimer's disease dementia
FTLD: Frontotemporal lobar degeneration
